# LASSO-derived model for the prediction of bleeding in aspirin users

**DOI:** 10.1038/s41598-024-63437-6

**Published:** 2024-05-31

**Authors:** Chen Liang, Lei Wanling, Wang Maofeng

**Affiliations:** 1 Department of General Surgery, Affiliated Dongyang Hospital, Wenzhou Medical University, Dongyang, 322100 Zhejiang China; 2 Department of Biomedical Sciences Laboratory, Affiliated Dongyang Hospital, Wenzhou Medical University, Dongyang, 322100 Zhejiang China

**Keywords:** Aspirin, Bleeding, Prediction model, Nomogram, Risk factor, Cardiology, Cardiovascular biology, Medical research, Biomarkers

## Abstract

Aspirin is widely used for both primary and secondary prevention of panvascular diseases, such as stroke and coronary heart disease (CHD). The optimal balance between reducing panvascular disease events and the potential increase in bleeding risk remains unclear. This study aimed to develop a predictive model specifically designed to assess bleeding risk in individuals using aspirin. A total of 58,415 individuals treated with aspirin were included in this study. Detailed data regarding patient demographics, clinical characteristics, comorbidities, medical history, and laboratory test results were collected from the Affiliated Dongyang Hospital of Wenzhou Medical University. The patients were randomly divided into two groups at a ratio of 7:3. The larger group was used for model development, while the smaller group was used for internal validation. To develop the prediction model, we employed least absolute shrinkage and selection operator (LASSO) regression followed by multivariate logistic regression. The performance of the model was assessed through metrics such as the area under the receiver operating characteristic (ROC) curve (AUC), calibration curves, and decision curve analysis (DCA). The LASSO-derived model employed in this study incorporated six variables, namely, sex, operation, previous bleeding, hemoglobin, platelet count, and cerebral infarction. It demonstrated excellent performance at predicting bleeding risk among aspirin users, with a high AUC of 0.866 (95% CI 0.857–0.874) in the training dataset and 0.861 (95% CI 0.848–0.875) in the test dataset. At a cutoff value of 0.047, the model achieved moderate sensitivity (83.0%) and specificity (73.9%). The calibration curve analysis revealed that the nomogram closely approximated the ideal curve, indicating good calibration. The DCA curve demonstrated a favorable clinical net benefit associated with the nomogram model. Our developed LASSO-derived predictive model has potential as an alternative tool for predicting bleeding in clinical settings.

## Introduction

Aspirin is widely used for primary and secondary prevention of cardiovascular events^1^. However, a notable challenge with aspirin therapy is the increased risk of bleeding complications in certain patients^2^. A crucial hurdle is to find the optimal balance between reducing cardiovascular events and minimizing bleeding risks^3^. Despite the proven efficacy of aspirin in preventing cardiovascular events, effectively managing the associated bleeding risk remains an ongoing challenge in clinical practice.

Several bleeding risk scores have been developed to help determine the best treatment regimen and duration. These studies have examined various aspects of bleeding risk assessment, providing valuable insights for clinical practice^4,5^. Previous investigations have established a straightforward risk score to identify high-risk individuals susceptible to primary upper gastrointestinal bleeding among aspirin users^6^. Recently, new clinical models have been developed and validated across diverse clinical scenarios, aiming to better predict hemorrhagic events. In clinical practice, a range of widely used scoring systems, such as BleeMACS^7^, TIMI risk score^8^, PARIS^9^, ARC-HBR^10^, CRUSADE^11^, ACUITY-GRACE score^11^, HAS-Bled score^12^, HORIZONS^13^, HAMPROW^14^, and CHA2DS2-VASC score^15^, are commonly used. These scores evaluate diverse clinical characteristics, including coronary anatomy, surgical interventions, genotyping, lifestyle factors, and adherence to treatment^16^.

In the era of precision medicine, incorporating tools such as the freely available clinical decision support tool (Aspirin-Guide) into electronic health records can enable personalized benefit-to-risk assessments for initiating aspirin therapy for primary prevention. The availability of various bleeding risk scores underscores the urgent need for a highly accurate clinical model capable of effectively adjusting the type and duration of aspirin use to minimize ischemic risk while avoiding an increase in bleeding risk. Each score has its own strengths and limitations, which depend on the characteristics of the patient cohorts used for development and validation, rendering them applicable only to specific patients, clinical contexts, and timeframes^16^. No risk score has achieved perfect predictive performance. Having an enhanced decision-making tool for evaluating bleeding risk in individuals would facilitate informed discussions with aspirin users.

To better predict bleeding risk in aspirin users, we developed and validated a novel risk prediction tool in this study.

## Methods

### Study population

Participants in this study were recruited from the Affiliated Dongyang Hospital of Wenzhou Medical University. The inclusion criterion for participants was documented use of aspirin recorded in the hospital's electronic medical records (EMRs) between January 2008 and December 2017. The exclusion criteria were missing aspirin data and a lack of relevant bleeding records. The study protocol received ethical approval from the Ethics Committee of the Affiliated Dongyang Hospital of Wenzhou Medical University (approval #2023-YX-408), and waived the requirement for the written informed consent of the patients All patient medical information was anonymized and deidentified before the analysis. This research involving human participants was conducted in accordance with the principles of the Declaration of Helsinki.

### Outcome definition

This study examined the occurrence of various types of bleeding, such as cerebral hemorrhage, gastrointestinal bleeding, mucosal bleeding, and other commonly observed bleeding events, within a 5-year period after the administration of aspirin. These incidents were identified through recorded data in the hospital's discharge EMRs. For the purpose of this study, the presence of any bleeding was classified as a positive outcome, while the absence of bleeding was considered a negative outcome.

### Risk factors

We extracted the following information from our hospital’s EMRs of the study participants: sex, age, height, weight, BMI, and past medical history, including smoking status, alcohol consumption status, diabetes status, hypertension status, surgical history, previous bleeding episodes, presence of tumors, acute myocardial infarction, percutaneous coronary intervention (PCI), presence of gastric ulcers, use of gastric protective drugs, presence of cerebral infarction, portal hypertension, anticoagulant usage, and various clinical test indicators, such as cardiac ejection fraction (EF), white blood cell count (WBC), platelet count (PLT), peripheral hemoglobin (HGB), and glomerular filtration rate (GFR). For the research parameters, we considered the lowest values of the clinical test indicators within one month before the initiation of aspirin. Other past medical histories were recorded if they occurred before the commencement of aspirin therapy.

### Data preprocessing

The data extracted from the clinical research big data platform underwent thorough cleaning procedures, which involved removing extreme values and imputing missing values. Indicators with missing values for over 20% of participants, such as height, weight, BMI, EF, and GFR, were excluded from the analysis. For the remaining predictor variables with missing values, multiple imputation techniques were applied. To train and evaluate the model, the data were divided into a training set comprising 70% of the data and a test set containing the remaining portion. The classification model was trained using the training set, while the performance of the model was assessed using the test set.

### Model building

The least absolute shrinkage and selection operator (LASSO) regression technique was employed to identify the most relevant predictive features^17^. The significant features identified through LASSO analysis were further subjected to stepwise backward multivariate logistic regression analysis. Based on this model, a nomogram was developed for predicting bleeding outcomes.

### Model evaluation

**Figure 1:**
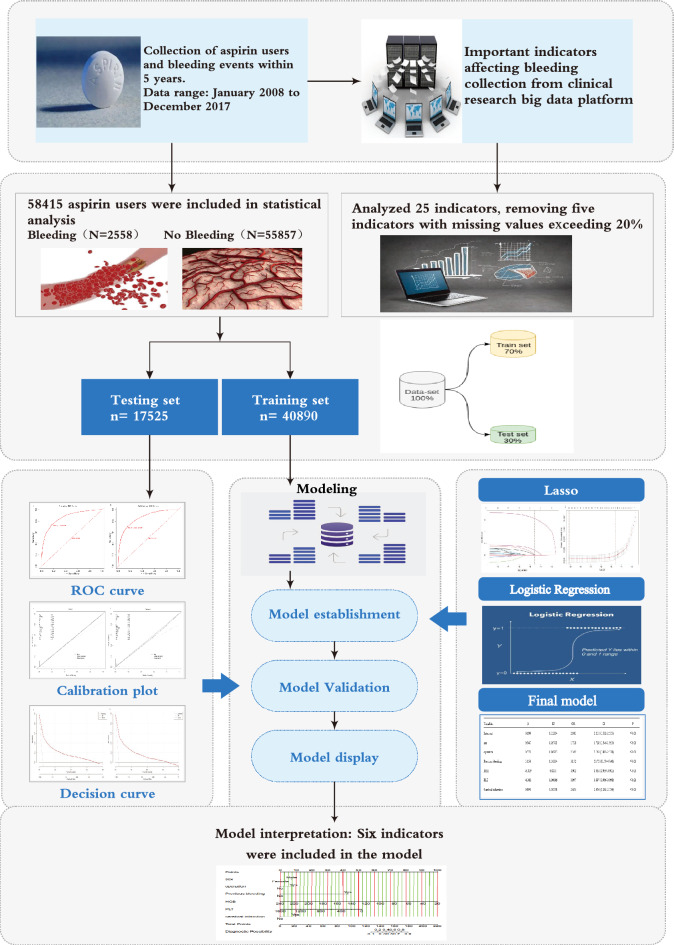
Study process flowchart.

The sensitivity and specificity of the model were evaluated as the area under the receiver operating characteristic (ROC) curve (AUC), which assesses a model’s discrimination performance. Calibration curves were analyzed to evaluate the agreement between the predicted and observed probabilities. The clinical effectiveness of the identified risk factors in predicting bleeding risk was verified through decision curve analysis (DCA), which considered the net benefit across different risk thresholds for patients. Moreover, the model's discriminatory performance was validated by comparing it with that of individual indicators. Figure 1 illustrates the flowchart depicting the process of model development and validation.

### Statistical methods

Statistical analysis and data visualization were performed using R4.2.1 software for Windows. Categorical variables are presented as n (%) and were compared using the χ^2^ test or Fisher’s exact test. Continuous variables are reported as mean ± standard deviation or median (interquartile range) and were compared using either Student's t test or the Mann‒Whitney *U* test. Multiple imputation techniques were implemented using the "mice" package. Baseline description and difference analysis were performed with the "comparegroups" package. LASSO regression was conducted using the "glmnet" package, while multivariable logistic regression was performed using the 'glm' function. Discrimination analysis was carried out using the "pROC," "ggROC," and "fbroc" packages. Calibration was assessed using the "rms" and "riskregression" packages. Decision curve analysis (DCA) was conducted using the 'rmda' package. The nomogram was created using the 'rms' package. Comparisons of multiple models for ROC analysis were conducted using the "ROCR" package. Diagnostic evaluation was performed with the "reportROC" package. All statistical tests were two-sided, with P < 0.05 considered statistically significant.

### Ethics approval

This study received approval from the Medical Ethics Committee of the Affiliated Dongyang Hospital of Wenzhou Medical University (approval #2023-YX-408). Informed consent was waived, and patient records/information were anonymized and deidentified before analysis.

## Results

### Study population characteristics

A total of 58,415 individuals who used aspirin were enrolled in this study, with 2558 experiencing bleeding events. Among the 25 variables assessed, only WBC, HGB, PLT, height, weight, BMI, EF, and GFR were continuous variables. Information on variables such as height, weight, BMI, EF, and GFR was missing for more than 20% of the patients, so these variables were excluded from the analysis. This left 20 variables with missing data for fewer than 20% of the patients (Appendix 1). All variables exhibited significant differences between the cohorts with and without bleeding. The baseline characteristics of the aspirin users are presented in Table 1. Patients were randomly allocated at a ratio of 7:3 to the training set (n = 40,890) or the test set (n = 17,525). Table 2 displays the baseline characteristics of patients in both sets, indicating no significant differences in any of the indicators between the two cohorts.

**Table 1:** Baseline Characteristics of subjects.

Variables	Total (N = 58,415)	No bleeding (N = 55,857)	Bleeding (N = 2558)	P value
Sex, n (%)				< 0.001^a^
Female	26,979 (46.19%)	26,008 (46.56%)	971 (37.96%)	
Male	31,436 (53.81%)	29,849 (53.44%)	1587 (62.04%)	
DAPT, n (%)				< 0.001^a^
No	40,007 (68.49%)	38,524 (68.97%)	1483 (57.97%)	
Yes	18,408 (31.51%)	17,333 (31.03%)	1075 (42.03%)	
Age (years)	63.0 [53.0;73.0]	63.0 [53.0;73.0]	69.0 [60.0;78.0]	< 0.001^b^
Smoker, n (%)				< 0.001^a^
No	29,888 (51.16%)	28,970 (51.86%)	918 (35.89%)	
Yes	28,527 (48.84%)	26,887 (48.14%)	1640 (64.11%)	
Drinker, n (%)				< 0.001^a^
No	29,888 (51.16%)	28,970 (51.86%)	918 (35.89%)	
Yes	28,527 (48.84%)	26,887 (48.14%)	1640 (64.11%)	
DM, n (%)				< 0.001^a^
No	52,223 (89.40%)	50,029 (89.57%)	2194 (85.77%)	
Yes	6192 (10.60%)	5828 (10.43%)	364 (14.23%)	
Hypertension, n (%)				< 0.001^a^
No	40,050 (68.56%)	38,736 (69.35%)	1314 (51.37%)	
Yes	18,365 (31.44%)	17,121 (30.65%)	1244 (48.63%)	
Operation, n (%)				< 0.001^a^
No	55,647 (95.26%)	53,410 (95.62%)	2237 (87.45%)	
Yes	2768 (4.74%)	2447 (4.38%)	321 (12.55%)	
Tumor, n (%)				< 0.001^a^
No	57,094 (97.74%)	54,636 (97.81%)	2458 (96.09%)	
Yes	1321 (2.26%)	1221 (2.19%)	100 (3.91%)	
Myocardial infarction, n (%)				0.014^a^
No	56,316 (96.41%)	53,873 (96.45%)	2443 (95.50%)	
Yes	2099 (3.59%)	1984 (3.55%)	115 (4.50%)	
PCI, n (%)				0.041^a^
No	58,166 (99.57%)	55,626 (99.59%)	2540 (99.30%)	
Yes	249 (0.43%)	231 (0.41%)	18 (0.70%)	
Previous bleeding, n (%)				0.000^a^
No	57,579 (98.57%)	55,560 (99.47%)	2019 (78.93%)	
Yes	836 (1.43%)	297 (0.53%)	539 (21.07%)	
WBC (10^9^/L)	5.15 [4.20; 6.25]	5.18 [4.24; 6.28]	4.50 [3.62; 5.53]	< 0.001^b^
HGB (g/L)	125 [111; 138]	126 [112; 139]	98.0 [76.0; 117]	0.000^b^
PLT (10^9^/L)	175 [138; 214]	176 [140; 215]	141 [103; 180]	< 0.001^b^
Gastric protective medicine, n (%)				< 0.001^a^
No	46,987 (80.44%)	45,291 (81.08%)	1696 (66.30%)	
Yes	11,428 (19.56%)	10,566 (18.92%)	862 (33.70%)	
Gastric ulcer, n (%)				< 0.001^a^
No	58,074 (99.42%)	55,578 (99.50%)	2496 (97.58%)	
Yes	341 (0.58%)	279 (0.50%)	62 (2.42%)	
Cerebral infarction, n (%)				< 0.001^a^
No	39,702 (67.97%)	38,634 (69.17%)	1068 (41.75%)	
Yes	18,713 (32.03%)	17,223 (30.83%)	1490 (58.25%)	
Portal hypertension, n (%)				< 0.001^a^
No	58,385 (99.95%)	55,838 (99.97%)	2547 (99.57%)	
Yes	30 (0.05%)	19 (0.03%)	11 (0.43%)	
Anticoagulants, n (%)				< 0.001^a^
No	42,346 (72.49%)	40,875 (73.18%)	1471 (57.51%)	
Yes	16,069 (27.51%)	14,982 (26.82%)	1087 (42.49%)	

*DAPT* dual antiplatelet therapy, *DM* diabetes mellitus, *PCI* percutaneous coronary intervention, *WBC* white blood cell count, *HGB* hemoglobin, *PLT* platelet count. ^a^Using the χ^2^ test or Fisher's exact test; ^b^Using Student's t test or the Mann‒Whitney *U* test.

**Table 2:** The baseline characteristics of the patients in the training and validation cohort.

Variables	Total N = 58,415	Validation N = 17,525	Training N = 40,890	P value
Sex, n (%)				0.557^a^
Female	26,979 (46.19%)	8061 (46.00%)	18,918 (46.27%)	
Male	31,436 (53.81%)	9464 (54.00%)	21,972 (53.73%)	
DAPT, n (%)				0.062^a^
No	40,007 (68.49%)	12,099 (69.04%)	27,908 (68.25%)	
Yes	18,408 (31.51%)	5426 (30.96%)	12,982 (31.75%)	
Age (years)	63.0 [53.0;73.0]	63.0 [53.0;73.0]	63.0 [53.0;73.0]	0.122^b^
Smoker, n (%)				0.234^a^
No	29,888 (51.16%)	9033 (51.54%)	20,855 (51.00%)	
Yes	28,527 (48.84%)	8492 (48.55%)	20,035 (49.00%)	
Drinker, n (%)				0.234^a^
No	29,888 (51.16%)	9033 (51.54%)	20,855 (51.00%)	
Yes	28,527 (48.84%)	8492 (48.55%)	20,035 (49.00%)	
DM, n (%)				0.580^a^
No	52,223 (89.40%)	15,648 (89.29%)	36,575 (89.45%)	
Yes	6192 (10.60%)	1877 (10.71%)	4315 (10.55%)	
Hypertension, n (%)				0.283^a^
No	40,050 (68.56%)	12,071 (68.88%)	27,979 (68.43%)	
Yes	18,365 (31.44%)	5454 (31.12%)	12,911 (31.57%)	
Operation, n (%)				0.309^a^
No	55,647 (95.26%)	16,719 (95.40%)	38,928 (95.20%)	
Yes	2768 (4.74%)	806 (4.60%)	1962 (4.80%)	
Tumor, n (%)				0.099^a^
No	57,094 (97.74%)	17,101 (97.58%)	39,993 (97.81%)	
Yes	1321 (2.26%)	424 (2.42%)	897 (2.19%)	
Myocardial infarction, n (%)				0.240^a^
No	56,316 (96.41%)	16,920 (96.55%)	39,396 (96.35%)	
Yes	2099 (3.59%)	605 (3.45%)	1494 (3.65%)	
PCI, n (%)				0.698^a^
No	58,166 (99.57%)	17,447 (99.55%)	40,719 (99.58%)	
Yes	249 (0.43%)	78 (0.45%)	171 (0.42%)	
Previous bleeding, n (%)				0.861^a^
No	57,579 (98.57%)	17,277 (98.58%)	40,302 (98.56%)	
Yes	836 (1.43%)	248 (1.42%)	588 (1.44%)	
WBC (10^9^/L)	5.15 [4.20; 6.25]	5.17 [4.21; 6.28]	5.14 [4.20; 6.24]	0.062^b^
HGB (g/L)	125 [111; 138]	125 [111; 138]	125 [111; 138]	0.592^b^
PLT (10^9^/L)	175 [138; 214]	175 [139; 215]	174 [138; 214]	0.171^b^
Gastric protective medicine, n (%)				0.767^a^
No	46,987 (80.44%)	14,110 (80.51%)	32,877 (80.40%)	
Yes	11,428 (19.56%)	3415 (19.49%)	8013 (19.60%)	
Gastric ulcer, n (%)				0.162^a^
No	58,074 (99.42%)	17,435 (99.49%)	40,639 (99.39%)	
Yes	341 (0.58%)	90 (0.51%)	251 (0.61%)	
Cerebral infarction, n (%)				0.648^a^
No	39,702 (67.97%)	11,935 (68.10%)	27,767 (67.91%)	
Yes	18,713 (32.03%)	5590 (31.90%)	13,123 (32.09%)	
Portal hypertension, n (%)				0.842^a^
No	58,385 (99.95%)	17,515 (99.94%)	40,870 (99.95%)	
Yes	30 (0.05%)	10 (0.06%)	20 (0.05%)	
Anticoagulants, n (%)				0.290^a^
No	42,346 (72.49%)	12,757 (72.79%)	29,589 (72.36%)	
Yes	16,069 (27.51%)	4768 (27.21%)	11,301 (27.64%)	

*DAPT* dual antiplatelet therapy, *DM* diabetes mellitus, *PCI* percutaneous coronary intervention, *WBC* white blood cell count, *HGB* hemoglobin, *PLT* platelet count. ^a^Using the χ^2^ test or Fisher's exact test; ^b^Using Student's t test or the Mann‒Whitney *U* test.

### Selected predictors and construction model

**Figure 2:**
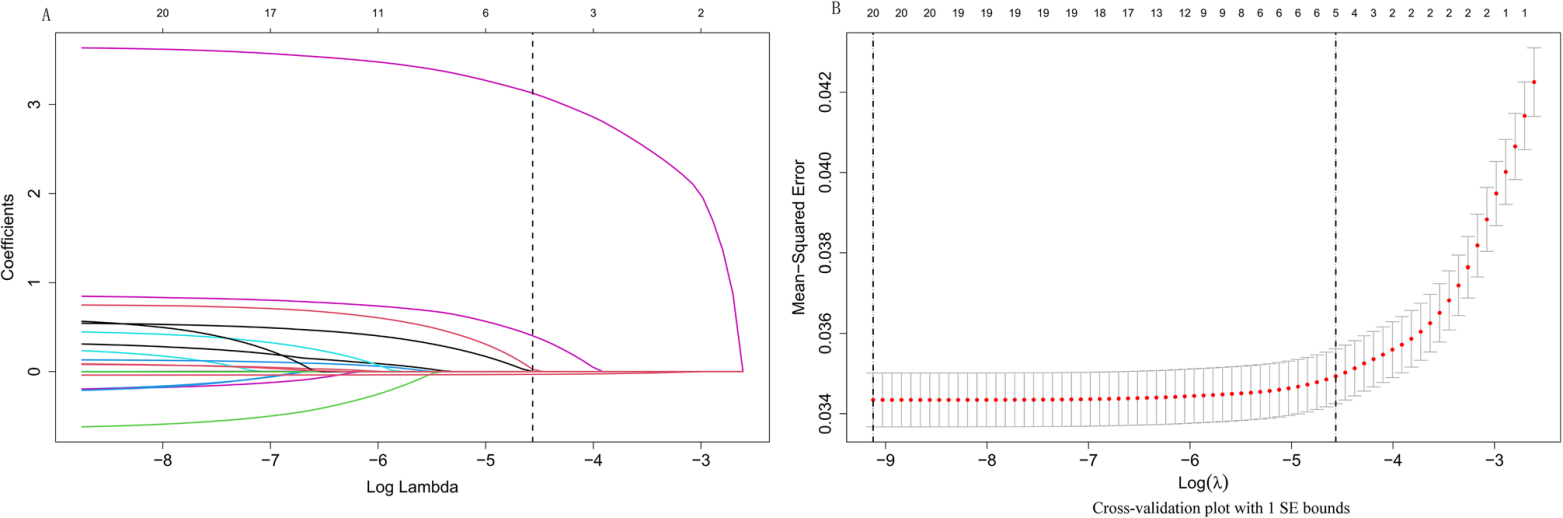
Variable selection was performed using LASSO regression. (**A**) Coefficient profile plots were generated by plotting the coefficients against the log(lambda) sequence. This visualization facilitated the variable selection process and enabled identification of the nonzero coefficient variables based on the optimal lambda value. (**B**) The optimal values, determined using the 1 standard error of the minimum criterion (lambda.1se), are represented by dotted vertical lines in the plots.

Following LASSO regression with tenfold cross-validation, six variables (sex, operation, previous bleeding, HGB, PLT, and cerebral infarction) were chosen for inclusion in the model based on the criteria of "family = binomial" and lambda.1se. The processes of variable shrinkage and cross-validation are depicted in Fig. 2A and B, respectively. Through multivariate logistic regression using the backward inclusion process, all six variables were retained in the final models (Table 3).

**Table 3:** Final model coefficients.

Variables	β	SE	OR	CI	P value
Intercept	0.699	0.122	2.011	2.011 (1.582–2.553)	< 0.001
Sex	0.547	0.057	1.728	1.728 (1.546–1.933)	< 0.001
Operation	0.771	0.087	2.162	2.161 (1.818–2.558)	< 0.001
Previous bleeding	3.656	0.103	38.72	38.720 (31.700–47.400)	< 0.001
HGB	− 0.039	0.001	0.962	0.961 (0.959–0.963)	< 0.001
PLT	− 0.003	0.001	0.997	0.997 (0.996–0.998)	< 0.001
Cerebral infarction	0.898	0.055	2.454	2.454 (2.202–2.736)	< 0.001

*HGB* hemoglobin, *PLT* platelet count.

### Model visualization

**Figure 3:**
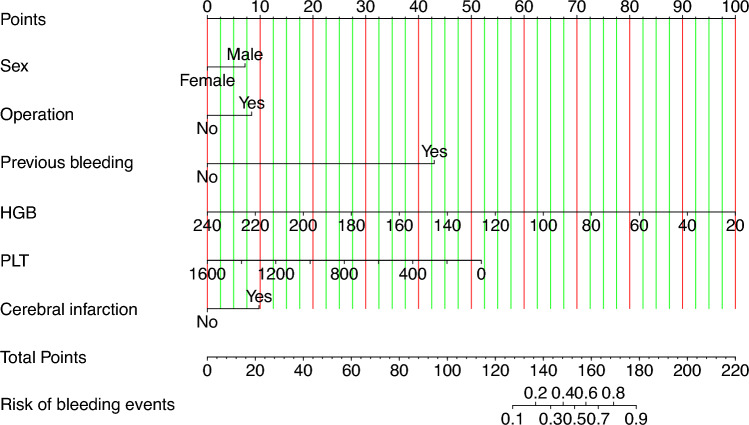
A nomogram based on the combination of six indicators was developed. If a patient's total score is 153, the corresponding probability of bleeding is 0.496. *HGB* hemoglobin, *PLT* platelet count.

The nomogram presented in Fig. 3 provides a visual tool for predicting the risk of bleeding in individuals using aspirin based on the results of logistic regression analysis. By identifying the value of each risk factor along the corresponding vertical line, the corresponding points can be determined. The total points are calculated by summing the points of the six risk factors. To predict the bleeding risk for a specific aspirin user, a vertical line is drawn from the total points axis, intersecting with the corresponding probability on the nomogram. For instance, if a female aspirin user has not undergone surgery, has no history of cerebral infarction, has experienced previous bleeding, and has a platelet count of 200 × 10^9^/L and an HGB level of 100 g/L, the total score would be 153. A vertical line drawn from the total score of 153 intersects the probability axis at 0.496, indicating an estimated probability of bleeding of 49.6%.

### Model validation

**Figure 4:**
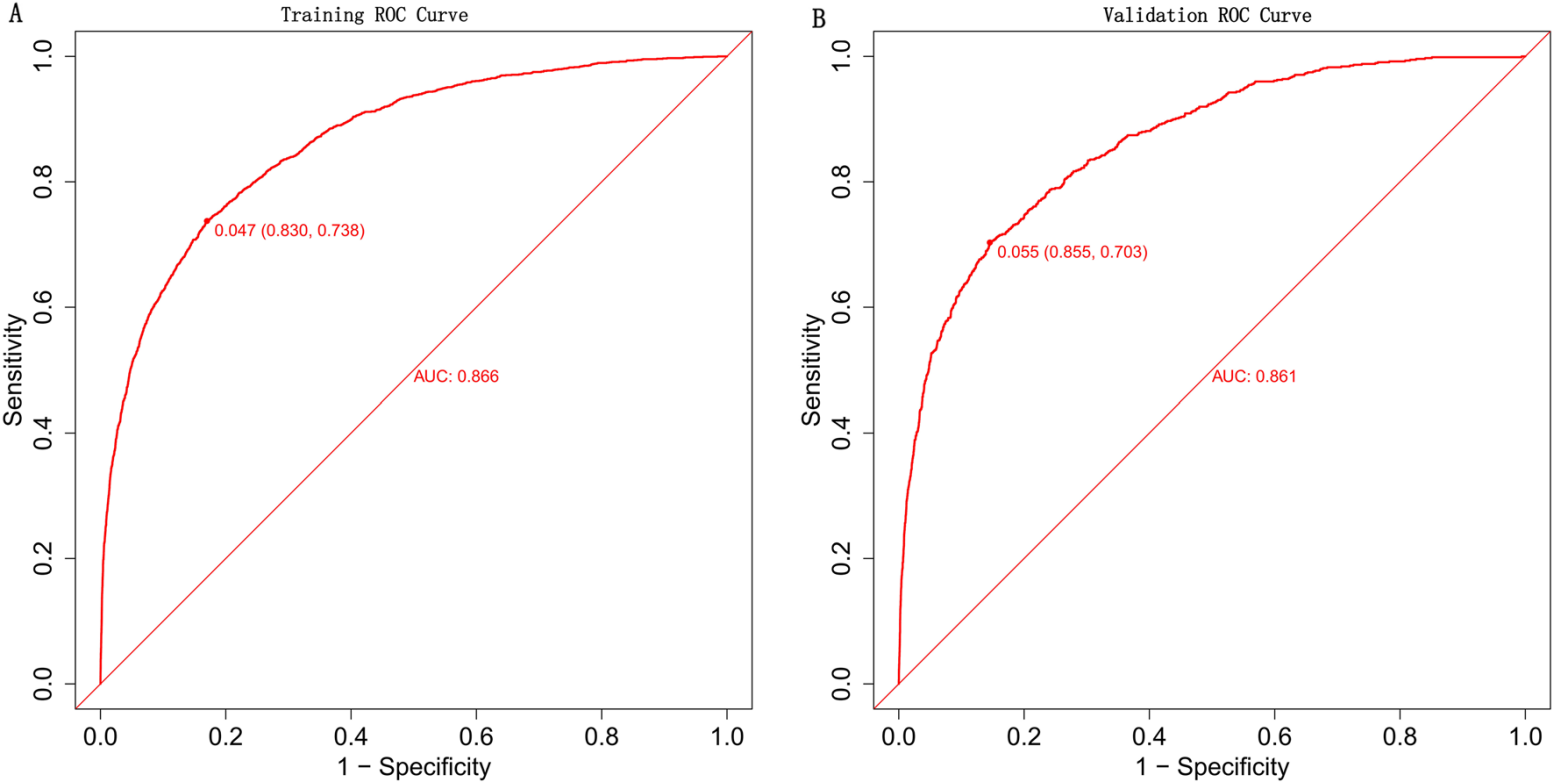
Receiver operating characteristic (ROC) curves of the model for distinguishing bleeding from nonbleeding patients. (**A**) Training set; (**B**) validation set. At a cutoff value of 0.047, the model achieved moderate sensitivity (83.0%), moderate specificity (73.9%), and a high negative predictive value (98.6%).

**Figure 5:**
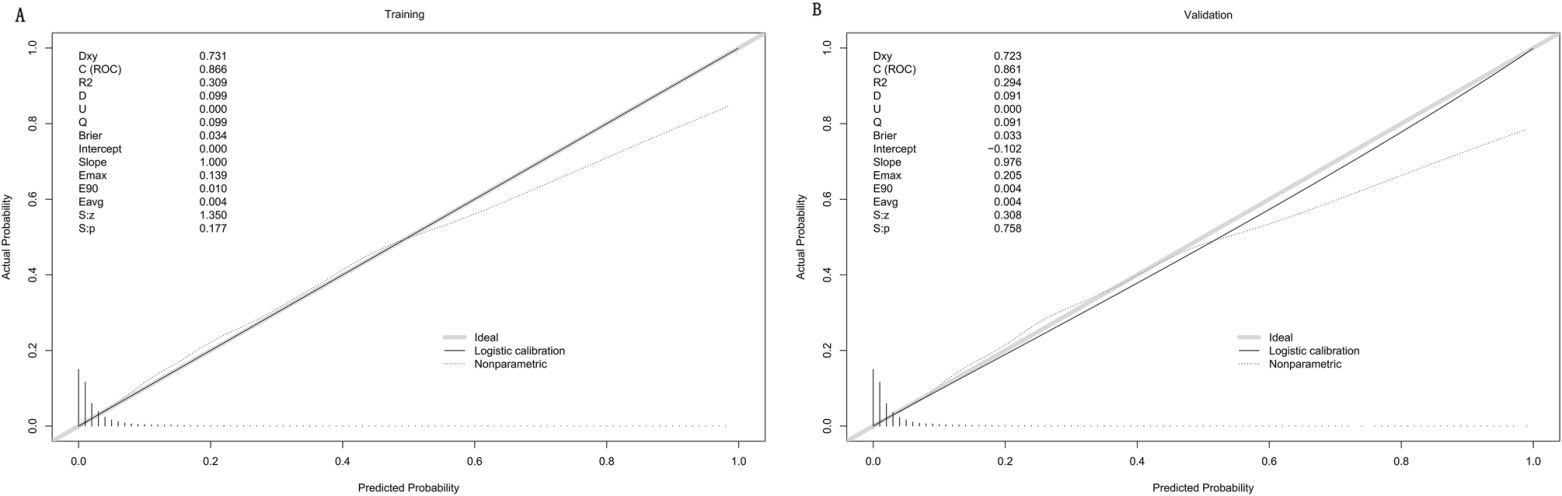
Calibration curves of the model. (**A**) Training set; (**B**) validation set. The y-axis represents the actual diagnosed cases of bleeding, while the x-axis represents the predicted risk of bleeding. Diagonal dotted lines were used to depict perfect predictions by an ideal model (gray line), while the black line represents the performance of the training set (left) and validation set (right). A closer alignment between the black lines and gray lines indicated better prediction performance.

**Figure 6:**
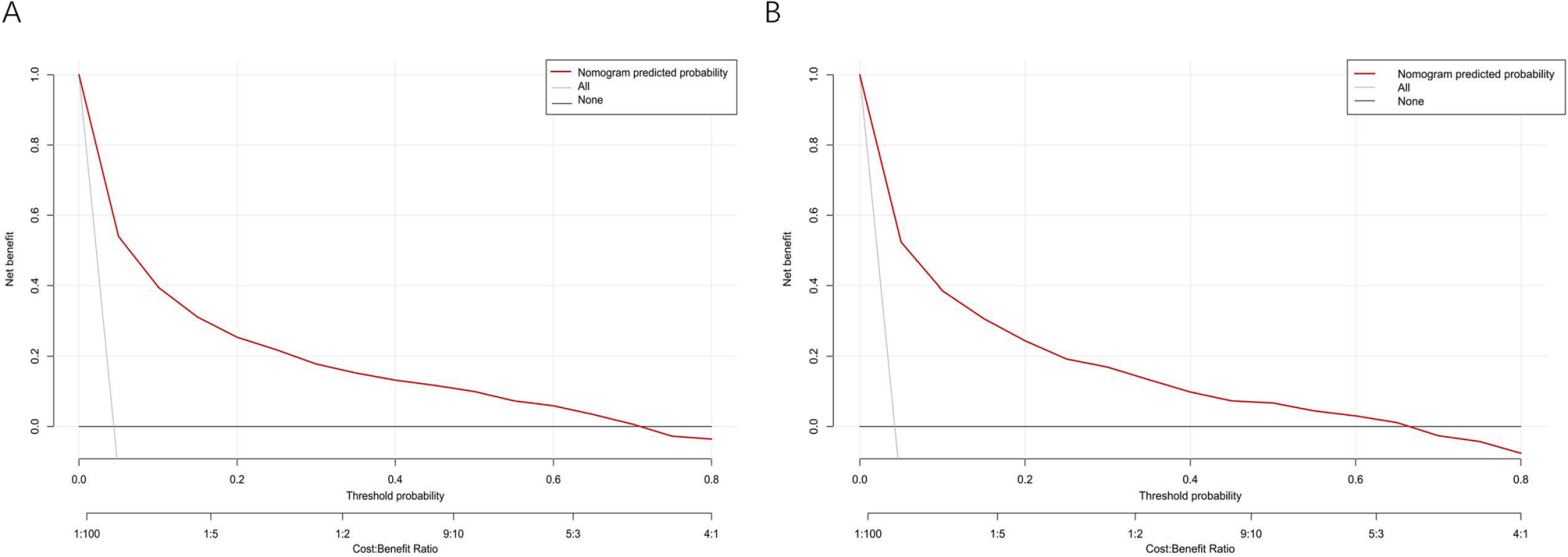
Decision curve analysis (DCA) of the model. (**A**) in the training set; (**B**) in the validation set. The y-axis of the graph represents the net benefit, while the horizontal lines labeled "None" indicate the assumption that no participant had bleeding. The lines labeled "All" represent the assumption that all participants had bleeding. The red lines correspond to the predictive model developed in this study.

To evaluate the discriminative ability of our model, we calculated the AUC of the ROC curve. Figure 4A shows that the AUC for the training dataset was 0.866 (95% CI 0.857–0.874), while Fig. 4B shows that the AUC for the test dataset was 0.861 (95% CI 0.848–0.875). By using a cutoff value of 0.047, the model achieved moderate sensitivity (83.0%), moderate specificity (73.9%), and a high negative predictive value (98.6%). The calibration curves, as shown in Fig. 5A and B, demonstrated excellent agreement between the predicted probability of bleeding and the actual observations in both the training and test sets. The Hosmer–Lemeshow goodness-of-fit (GOF) test also supported good consistency (p = 0.089). Figure 6 illustrates the results of decision curve analysis (DCA) for our developed model. The DCA results indicated a favorable net benefit in predicting bleeding risk among aspirin users. The threshold probability ranges were 1.0–72% for the training dataset (Fig. 6A) and 1.0–68% for the test dataset (Fig. 6B).

### Model comparison with a single indicator

**Figure 7:**
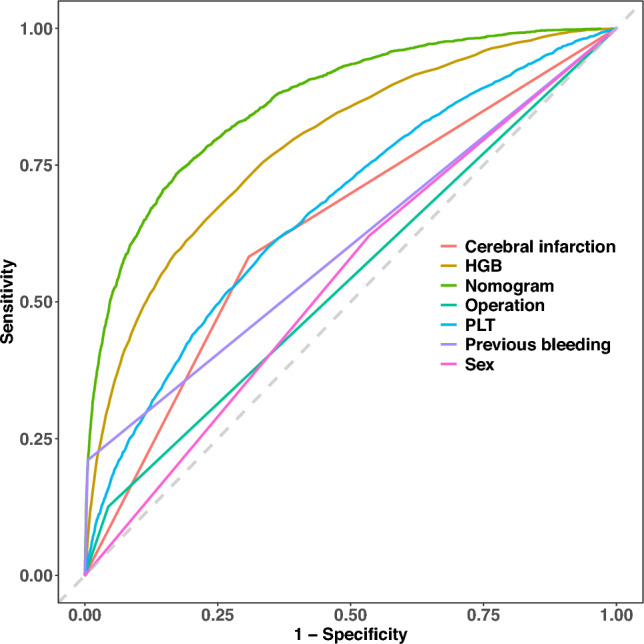
Comparison between the nomogram and individual indicators. *HGB* hemoglobin, *PLT* platelet count.

We assessed the discriminative capability of our developed model (nomogram) compared to that of different single indicators. Figure 7 clearly shows that our model outperformed the single indicators in terms of discriminative ability.

## Discussion

In this study, we developed and validated a LASSO-derived model to evaluate the risk of bleeding in individuals taking aspirin. The final model included six significant predictors: sex, past surgery, previous bleeding, HGB, PLT, and cerebral infarction. Our model exhibited excellent discrimination, calibration, and net clinical benefit. The nomogram provides an intuitive graphical representation of the results, serving as a valuable tool for clinicians to estimate bleeding risk in aspirin users.

Various factors have been reported to affect bleeding in individuals using aspirin, including HGB^18^, PLT^19,20^, previous bleeding^18,21^, cerebral infarction^22,23^, sex^24^, and past surgery^25^. Our objective was to incorporate this comprehensive information into our risk models for predicting bleeding. Previous studies have emphasized the significance of lower HGB as a strong predictor of major bleeding^26^, as it has been linked to higher bleeding rates^27^. Our study reaffirmed the critical role of HGB as an indicator for assessing bleeding risk. We also identified PLT count as another important predictor, where lower PLT was associated with higher bleeding risk and poorer prognosis^28^. Our predictive model aligns with the findings of previous studies. A history of bleeding has been established as a crucial factor in guiding treatment plans for aspirin users^18^. Our risk model demonstrated that previous bleeding increases the risk of bleeding in aspirin users. Artery occlusion cerebral infarction has been associated with a heightened risk of hemorrhage transformation^29^, which is consistent with our results and may be attributed to increased use of antithrombotic medications. Our finding that male sex was independently associated with higher bleeding risk is also consistent with earlier research^30^. In addition to the aforementioned factors, a history of surgery has been recognized as a potential predictor of bleeding^31^, and our findings support the significant association between surgical history and elevated bleeding risk. In clinical practice, taking aspirin in conjunction with anticoagulants or undergoing dual antiplatelet therapy (DAPT) has a synergistic effect that greatly increases the risk of bleeding events. However, our prediction model does not include markers for anticoagulants or DAPT. This omission may be attributed to the fact that in our model, previous bleeding serves as a crucial indicator. Furthermore, medical professionals exercise greater caution when considering the use of DAPT and anticoagulants in patients with a history of previous bleeding.

Tailored management strategies are essential for aspirin users with different levels of bleeding risk. High-risk patients require a comprehensive evaluation and optimization of their condition before prescribing aspirin. The development of an accurate predictive model for bleeding in aspirin users has significant clinical implications. Such a model can aid in managing comorbidities, optimizing blood parameters (e.g., HGB, PLT), considering clinical features (e.g., age, sex), addressing underlying diseases (e.g., operation, previous bleeding), and evaluating the patient’s medications to improve coagulation function. The model's results are presented in a user-friendly nomogram, letting clinicians easily estimate individual bleeding risk and make personalized treatment decisions. Evaluation metrics, including the clinical decision curve, demonstrate a high net clinical benefit, indicating the potential for improved patient outcomes and reduced healthcare costs.

Despite the notable findings of our study, it is important to acknowledge certain limitations. First, the retrospective design of our study prevents us from establishing causal relationships. Further validation through prospective studies is warranted to confirm the predictions of the model developed here. Second, the presence of missing data for certain variables may introduce bias; however, we addressed this bias by excluding variables with substantial missing information (> 20% of patients). Moreover, our study specifically focused on aspirin users, and the generalizability of our findings to other patient populations requires additional investigation.

Overall, our model provides valuable clinical insights and supports decision-making in managing bleeding among aspirin users, potentially optimizing treatment planning, improving patient outcomes, and enhancing resource allocation. Future research should externally validate our model in diverse patient cohorts to ensure its reliability and effectiveness in routine clinical practice.

### Supplementary Information

Supplementary Information.

## Data Availability

The data that support the findings of this study are not openly available due to reasons of sensitivity and are available from the corresponding author upon reasonable request. Data are located in controlled access data storage at the Affiliated Dongyang Hospital of Wenzhou Medical University.
