# Supplementary figures and images for: LASSO-derived model for the prediction of bleeding in aspirin users
Source: Sci Rep. 2024 May 31;14:12507. doi: 10.1038/s41598-024-63437-6 (PMC11143346; doi:10.1038/s41598-024-63437-6)

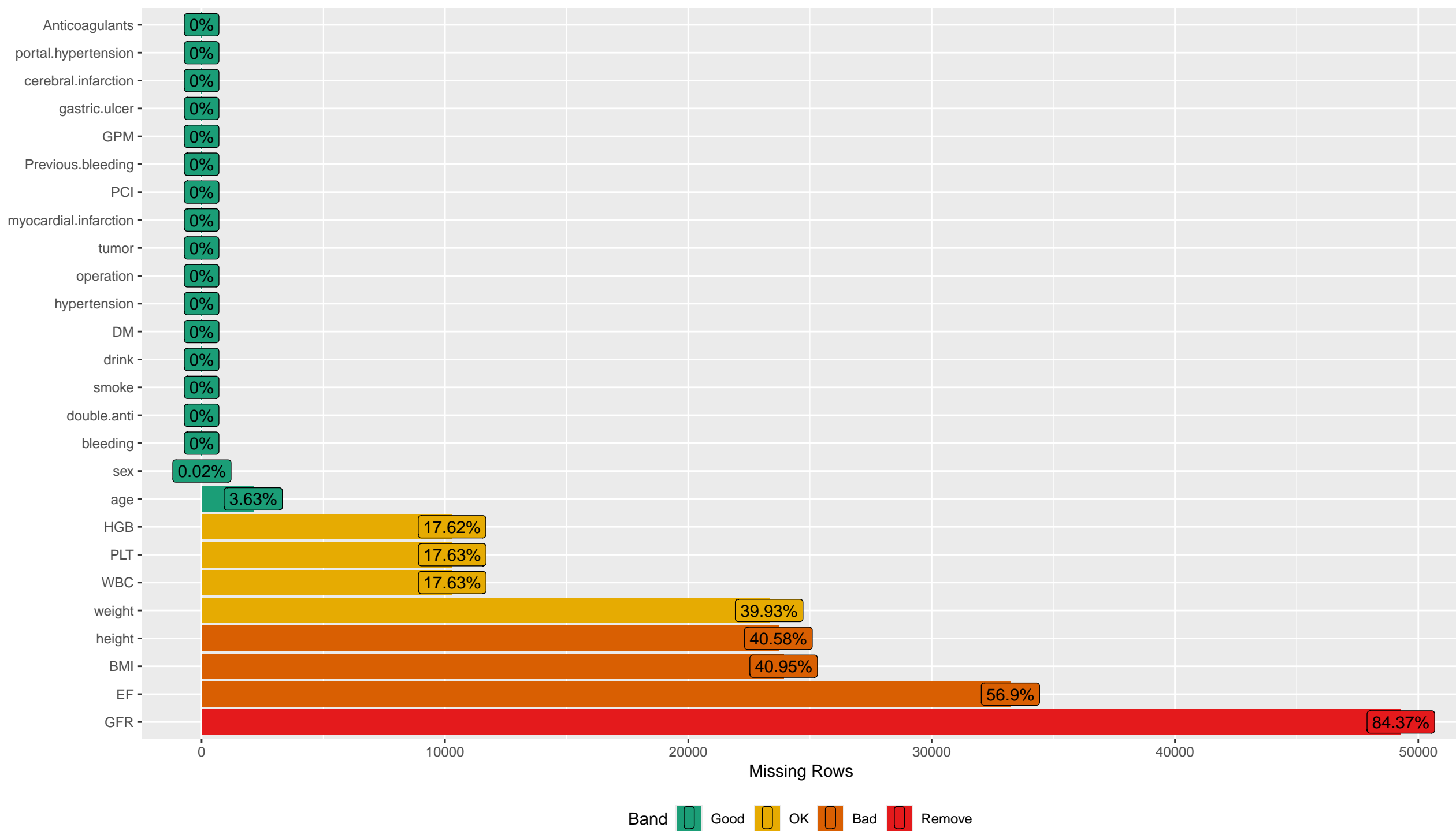

Supplement: Supplementary file 1 — Supplementary Information. [file 41598_2024_63437_MOESM1_ESM.pdf]
